# Supplementary material for: Vertigo in Patients with Degenerative Cervical Myelopathy
Source: J Clin Med. 2021 Jun 4;10(11):2496. doi: 10.3390/jcm10112496 (PMC8201049; doi:10.3390/jcm10112496)
Supplement: Supplementary file 1 [file jcm-10-02496-s001.zip › jcm-1225161-supplementary.pdf]

1. Have you suffered from dizziness or vertigo during the last six months? Y/N

2. If YES, specify further the type of vertigo:

   2.1. spinning vertigo, as if on a carrousel (**spinning vertigo**) Y/N

   2.2. swaying vertigo, as if on a small boat (**swaying vertigo**) Y/N

   2.3. feeling of impending black-out when standing up rapidly (**orthostatic vertigo**) Y/N

   2.4. none of these three types (**unspecified dizziness**) Y/N

3. If YES, is the vertigo:

   3.1. triggered or aggravated by head movements? Y/N

   3.2. triggered by a change of position (e.g. standing up from lying down?) Y/N

   3.3. also present when sitting or lying down? Y/N

   3.4. only present when standing or walking? Y/N
